# At the Crossroads of Minimally Invasive Mitral Valve Surgery—Benching Single Hospital Experience to a National Registry: A Plea for Risk Management Technology

**DOI:** 10.3390/jcdd9080261

**Published:** 2022-08-11

**Authors:** Riccardo Cocchieri, Bertus van de Wetering, Sjoerd van Tuijl, Iman Mousavi, Robert Riezebos, Bastian de Mol

**Affiliations:** 1 Cardiothoracic Surgeon, OLVG Hospital, 1091 AC Amsterdam, The Netherlands; 2 Biomedical Engineer, LifeTec Group BV, 5611 ZS Eindhoven, The Netherlands; 3 Cardiothoracic Surgery Resident, OLVG Hospital, 1091 AC Amsterdam, The Netherlands; 4 Cardiologist, OLVG Hospital, 1091 AC Amsterdam, The Netherlands; 5 Department of Cardiothoracic Surgery, Amsterdam University Medical Center, 1105 AZ Amsterdam, The Netherlands; 6 Department of Biomedical Engineering, Eindhoven University of Technology, 5600 MB Eindhoven, The Netherlands

**Keywords:** risk management, risk management methods, minimally invasive mitral valve surgery, surgical training, simulator training

## Abstract

Almost 30 years after the first endoscopic mitral valve repair, Minimally Invasive Mitral Valve Surgery (MIMVS) has become the standard at many institutions due to optimal clinical results and fast recovery. The question that arises is can already good results be further improved by an Institutional Risk Management Performance (IRMP) system in decreasing risks in minimally invasive mitral valve surgery (MIMVS)? As of yet, there are no reports on IRMP and learning systems in the literature. (2) *Methods*: We described and appraised our five-year single institutional experience with MIMVS in isolated valve surgery included in the Netherlands Heart Registry (NHR) and investigated root causes of high-impact complications. (3) *Results*: The 120-day and 12-month mortality were 1.1% and 1.9%, respectively, compared to the average of 4.3% and 5.3% reported in the NHR. The regurgitation rate was 1.4% compared to 5.2% nationwide. The few high-impact complications appeared not to be preventable. (4) *Discussion*: In MIMVS, freedom from major and minor complications is a strong indicator of an effective IRMP but remains concealed from physicians and patients, despite its relevance to shared decision making. Innovation adds to the complexity of MIMVS and challenges surgical competence. An IRMP system may detect and control new risks earlier. (5) *Conclusion*: An IRMP system contributes to an effective reduction of risks, pain and discomfort; provides relevant input for shared decision making; and warrants the safe introduction of new technology. Crossroads conclusions: investment in machine learning and AI for an effective IRMP system is recommended and the roles for commanding and operating surgeons should be considered.

## 1. Background

It is recommended that surgery should be reserved for primary mitral valve disease, and transcutaneous interventions for inoperable primary and secondary mitral valve disease in which medical treatment falls short [1].

In functional mitral valve disease, the effectiveness of, and prognosis for the reduction of mitral valve regurgitation (MVR) are determined by their etiology, recovery potential and the degree of severity of disease. From this perspective, the indications and contraindications for minimally invasive mitral valve surgery (MIVMS) and transcatheter mitral valve repair (TMVR) for the treatment of functional MVR are similar. The choice between MIMVS or TMVR depends on the interventional risk and the expected completeness of reduction of MVR. Today, in properly selected patients interventional risks are low in both MIMVS and TMVR alike. This raises the question of what makes new technologies contribute to further improving outcomes in the treatment of MVR. There is no doubt that the potential of TMVR and improved diagnostic work-up has revived the interest in the treatment of MVR, which in turn has resulted in more patients becoming eligible for intervention [2] with TMVR or MIMVS.

The risk profile of the individual patient and the risk profile of the intervention may favor either MIMVS or TMVR or medical treatment. To this end, not only a sufficiently detailed risk profile of the individual patient is required, but also the compilation of a risk profile for the procedure and pre- and postoperative risk management by the particular institution, i.e., the institution’s risk management performance (IRMP).

The nature of the risks of high-impact complications such as death, stroke, myocardial infarction and sepsis can be devastating and may seriously affect prognosis and quality of life, even if the intervention is successful. Surgery in general is responsible for the majority of in-hospital adverse events [3,4]. Considering the impact on the patient and their social environment, the risks for each individual patient should be kept as low as reasonably possible. Only when this principle is implemented should one speak of a residual risk or an acceptable complication. The need for the critical adjudication of the term low-risk has been emphasized by recent reports stating that in retrospect, mortality in low-risk cardiac surgery could have been prevented in a substantial number of patients [5,6]. Therefore, the established low-risk performance of MIMVS and TMVR should not limit efforts to further decrease interventional and clinical risks. An assessment of indications, benefits, procedural risks and pitfalls, institutional experience and outcomes of MIVMS should also minimize debate about fear of pain and suffering. When optimally informed about benefits and risks, the patient’s wishes should prevail in a process of shared decision making.

Comparing results in a registry may reveal better outcomes for a particular institution, which should lead to a second look and to questioning how this was achieved, the degree to which selection bias played a role, or if there was indeed an effective risk reduction or IRMP system in place. We should also ask ourselves if this finding is the result of a particular surgical approach and if there is still room for improvement, and if so by what means? Should we invest in more innovative tools and devices or in better learning from experience and risk management for improving work processes? Even when results of MIMVS appear to be very good, there could be a potential for improvement.

It is important to keep in mind that the success of TMVR is based on the simplification of the implantation process, despite the limitations of repair possibilities. Process-wise, transapical off-pump procedures for neochordae implantation exclude the risks associated with extracorporeal circulation and median sternotomy. The trade-off can be limitations of the repair and new risks, including a learning curve [7]. Therefore, MIMVS primarily focuses on managing indications and risks.

In this contribution, we analyzed our single institution’s 5-year experience (2016–2020) with MIMVS for isolated valve disease as reported in the Netherlands Heart Registry (NHR) [8]. We described the extend to which risk analysis methods to find the root causes of failures and unwanted events are available. From the risk control perspective, we also explored those technologies which could be promising for improving IRMP and may change MIMVS performance to the lowest acceptable risk. We hypothesized that MIMVS has much to gain from technologies for patient management and risk control. In TMVR, which is a simpler process, there is much to be gained by predicting the effect of the intervention by means of computer modeling and advanced imaging.

## 2. Methods

1. The results of MIMVS performed at the OLVG hospital were compared with the results for isolated mitral valve surgery from other institutions reported in the NHR; this included both conventional surgery and MIMVS. The root cause of serious adverse events was analyzed by means of Phases of Care Mortality Analysis (POCMA) for low-risk cardiac surgery [6,9]. 

2. We presented useful reports from the literature on risk analysis methods in surgery, with a focus on cardiac surgery. The methods we searched for were required to provide a root cause analysis resulting in preventive and risk-mitigating actions. We also explored how promising biomedical engineering solutions, imaging and computational technologies may improve the safety of MIMVS interventions. 

## 3. Results

### 3.1. Institutional Risk Management Performance

**Figure 1:**
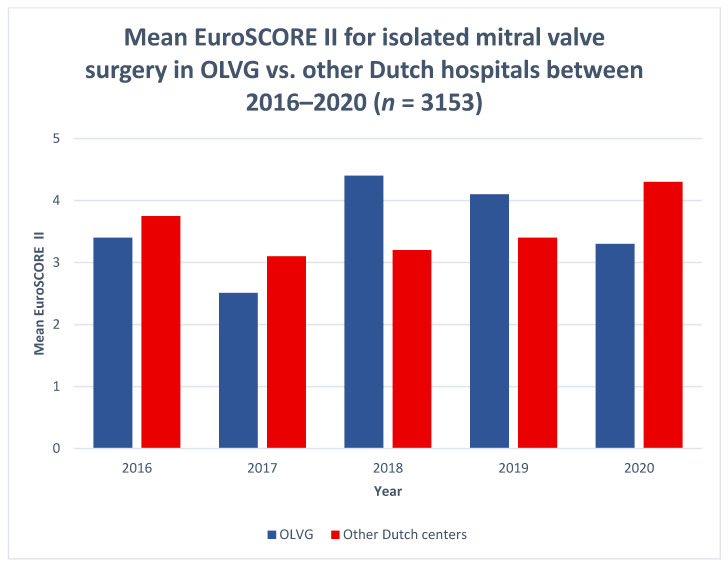
Comparison between the OLVG preoperative mean EuroSCORE II vs. other Dutch hospitals.

In the five-year period of 2016–2020, 3613 patients underwent open-heart surgery at the OLVG Hospital, Amsterdam, the Netherlands. During this period, 395 patients had some form of combined MIMVS surgery and 372 patients underwent isolated mitral valve replacement, 99% of which were cases of MIMVS. The rate of patients with a high EuroSCORE II of 9.5 or more was 5.6 for the OLVG group, and 9.4% for the NHR group, but the average EuroSCORE II in the 5-year period was similar (Figure 1). The mean EuroSCORE II of the OLVG MIMVS group was 3.56%. See Table 1. [8]. The 120-day and 1-year mortality rates were 1.1% and 1.9%, respectively, compared with 4.3% and 5.3 % for isolated mitral valve surgery registered for the OLVG and entire NHR group. The rate of OLVG postoperative mitral regurgitation was 1.4%, and in the NHR group this was 5.2%. The rates of re-exploration for bleeding, deep sternal wound infection and CVA within 30 postoperative days were 2.2%, 0.0% and 0.3%, respectively, and this was 7.1%, 0.4%, and 1.2% for the NHR group. The 5-year survival was within the 95% confidence bands for the entire NHR population.

We retrospectively analyzed root causes and initiating events for major complications such as mortality, re-exploration for bleeding, serious stroke and all-cause heart failure. The results of our findings are presented in Table 2 and Table 3**.**

### 3.2. Anticipating Risks and Learning by Experience

There are several methods of improving risk management by using the feedback results obtained from the root cause analysis of a particular adverse event, or a cluster of events. Based on the literature, the following are the most commonly available methods that can be applied to MIMVS.

#### 3.2.1. Prevention and Recovery Information System for Monitoring and Analysis

Driessen and colleagues recently presented a literature review on the use of the Prevention and Recovery Information System for Monitoring and Analysis (PRISMA) method for health care institutions [10]. The application of PRISMA provides a root cause analysis of factors such as unplanned ICU admissions [11]. Due to its medical and logistical complexity, the rate of unintended harm is higher in surgical care. A study in the Netherlands that included 10 surgical units applied PRISMA to the root cause analysis of the unintended events, 33.0 percent of which were found to be medication errors. However, most root causes were human error (72.3%), followed by organizational (16.1%) and technical errors (5.7%) [3]. In a more extensive surgical study, 41% of complications were considered to be preventable [4]. The PRISMA method relies on event reporting and a systematic review of patient records. PRISMA has also been applied to determine the reasons for the common nuisance of equipment-related incidents in the operating room [12].

#### 3.2.2. Phases of Care Mortality Analysis

The Phases of Care Mortality Analysis (POCMA) method of assessment was adopted by the Society of Thoracic Surgeons (STS) for cardiac surgical procedures after its initial introduction by the Michigan Society of Thoracic and Cardiovascular Surgeons as a method of analyzing mortality and quality improvement [6,13]. In the POCMA system, deaths and complications are sorted according to occurrence in one of the following five main process phases of surgery: preoperative, intraoperative, ICU, floor, and discharge. Each phase has specific subcategories such as anesthesia, surgeon, cardiopulmonary bypass, and catastrophe. The adjudication is conducted by a dedicated team. Although initiated to analyze mortality, POCMA has also been used to investigate comparative mortality cases such as the difference in mortality between TAVR and surgical AVR, and the difference in mortality rate between high-volume and low-volume case surgeons in the surgery of acute type A dissection [14,15]. POCMA also provides a root cause analysis as well as an estimation of the magnitude of the risk [16,17]. Liden K et al. and Mejia OA et al. applied POCMA to low-risk cardiac surgery patients and concluded that even low-risks of mortality were avoidable [5,6].

#### 3.2.3. Failure Mode and Effects Analysis and Fault Tree Analysis

Failure Mode and Effects Analysis (FMEA) has multiple applications. It can be applied to the design and manufacturing and use of devices. Other uses include the administration of drugs in a particular setting or MRI in the presence of implantable cardiac devices [18,19]. FMEA involves listing all known risks based on experience and reports from the literature. These are then discussed by a panel of experts and stakeholders and the rate of occurrence and severity of harm are subsequently assessed. Based on a calibrated scale specific to the activity, the weight of the risk is defined by a formula which yields a so-called risk priority number (RPN). This number relates to a scale which defines a risk as low, medium or severe (green, orange or red). The analysis must include risk mitigation actions and also indicate by how many points the weight of the risk is expected to be reduced. Follow-up should then confirm that the assumptions made in the FMEA are either realistic or that they need to be adjusted. The use of FMEA prioritizes failure modes in an absolute and comparative way, depending on what is defined as an acceptable risk. Using FMEA elucidates the complexity of certain processes in surgery. Apart from prioritizing risks and defining mitigation actions, it can also result in the redesigning of a process.

A prospective analysis of operating room procedures with FMEA identified 10 important processes, seven subprocesses, and 187 failure modes, 36 of which were marked as high-risk failures [20]. The application of FMEA to the transfer of patients from the Operating room to the Intensive Care Unit revealed 79 process failures in 37 individual steps, and 22 high-risk failures [21]. From this perspective, applying FMEA to TMVR interventions has indeed resulted in a drastic redesign and simplification of the logistical chain.

Fault Tree Analysis (FTA) is very commonly used in high-reliability, high-risk, low public-tolerance industrial activities such as power plants, aviation and sometimes the medical device industry. Although the analysis input is provided by a team of experts, the graphical display allows multibranch and hierarchical presentation of contributing factors. It provides a top-down event analysis. Ruiters and Stoelinga provided a useful overview of the applications, which—thanks to software—may explain how failures are propagated through the system, and how component failures can disrupt complex work processes [22]. A strong feature of FTA is that it tests the reliability of a system or a work process design in terms of preventing harm, or to mitigate the effects of failure. For a simple example see an instructive tutorial on https://www.youtube.com/watch?v=dKtIG0UXS6Y (accessed on 9 August 2022).

In summary, there are various methods for learning and root cause analysis. What they have in common is that stakeholders analyze the event in a structured way by identifying the risks and assessing if these risks were detected and mitigated effectively. PRISMA and FMEA/FTA include precursor events and continuously monitor if the actual event analysis matches the initial risk assumptions. A risk management target or a discovery objective must be defined. A narrow focus on individual performance or a wide focus on institutional mortality rates may overlook essential shortcomings in the system or particular processes. This prevents effective risk mitigation and may even wrongly place blame on an institution [23]. Last but not least, the aforementioned risk-control tools are relatively time-consuming and demand input from data specialists [24], quality control experts [3,21], and human error engineers [25].

### 3.3. Risk Reduction by Patient Management and New Technologies

Four types of MIMVS innovation can be identified and are as follows: 1. access to and availability of surgical tools; 2. concepts and devices for patient management; 3. imaging-based planning tools; 4. computational modeling, and data management for learning.

#### 3.3.1. Access and Tools

Better tools and smarter work processes should result in fewer complications and higher success rates. It has proved virtually impossible to conduct a study that can provide evidence that the availability of a single or even a number of ‘smart’ tools indeed improves the clinical outcome of cardiac surgery. The auxiliary technology of MIMVS makes the process easier. Robotic-assisted surgery is promoted as being the least-invasive form and clinical reports boast good results, although head-to-head comparative studies on MIMVS are missing [26,27]. There are also recommendations for caution [28]. In MIMVS, the devil is in the details, and special care is called for in the administration of cardioplegia [29], and optimal venous drainage [30]. Additionally, in a comparative study between open-sternum and robotic-assisted mitral valve surgery, the myocardium was found to remain warmer for a longer time in robotic surgery [31]. Some authors label the repair of MVR by the transapical implantation of a neochordae, an off-pump in a beating heart as ‘micro-invasive’ surgery, which has proven safe and effective, although the results are limited [7,32]. Van Praet et al. argue that MIVMS should be part of a minimally invasive program [33] including a dedicated heart team [34], and that this should also be applicable to AVR and CABG [35,36,37] in which preparedness, planning of procedures and the role played by a multidisciplinary heart team are all pivotal.

#### 3.3.2. Patient Management

Active perioperative risk management can prevent complications. Closure of the left atrial appendage reduces the risk of early and late postoperative stroke in patients with atrial fibrillation [38,39]. In surgery and transcutaneous procedures, a vascular closure device is safe to use and allows for the percutaneous cannulation of the femoral vessels [40]. Verstraete et al. in 2021 provided a useful review with a variety of considerations for antithrombotic treatment after surgery and transcutaneous procedures [41,42]. The use of direct oral anticoagulants (DOACs) is the subject of ongoing studies, although occasional studies have shown MIMVS to have promising results [43]. Pain relief is essential for enjoying the benefits of minimal access, and interventions under regional and local anesthesia can be considered [44,45]. Postoperative delirium can seriously delay postoperative recovery despite patients having undergone MIMVS or a transcutaneous procedure. Patients at risk should be identified and monitored for well-known risk factors both pre- and postoperatively [46,47]. A simple disposable detection device is available to assist in the postoperative monitoring and early recognition of symptoms of delirium by means of performing a 1 min single-channel EEG [48]. Last but not least, recovery should be embedded in a patient-tailored rehabilitation program [49,50] specifically designed to assist the patient to gain the best advantage from the surgery. However, although technology and patient management reduce risks, they also have downsides such as extra costs, results being under investigation and new risks. See Table 4.

#### 3.3.3. Imaging and Planning

Full preoperative multimodality imaging has been recommended as the best of the many available options for treating a complex disease such as MVR. [51]. Preoperative planning may include 3-D reconstruction of a patient’s anatomy, rapid prototyping and computational modeling [52,53,54] for both MIVS and TMVR, including transcutaneous mitral valve replacement [55]. The availability of transcutaneous solutions has spurred efforts by biomedical engineers to simulate the complexity of the mitral valve by means of a blueprint [56,57], as well as the impact of the intervention [58,59] including determining the length of the neochordae [60,61].

#### 3.3.4. Data Management, Feedback and Learning

Medical decision making and risk assessment in particular also require data from sources other than imaging. These include a national registry, the literature and institutional experience. Apart from the incompleteness of registry data, biases of various kinds may affect an objective assessment of risks [62]. This may not only affect the quality of the risk information, but also result in the selective use of institutional experience. Artificial intelligence and deep learning can provide performance feedback and predictions at various levels ranging from the individual surgeon to institution and national professional standards [63,64,65].

## 4. Discussion

First of all, every patient is entitled to receive all relevant information regarding the benefits and risks of a procedure. Shared decision making is essential as the patient may attach special significance to their very personal and social circumstances [66,67,68,69,70]. The patient has to estimate their own ability to tolerate the procedure and to test their resilience in coping with disappointments and adverse events. *Frailty* can be mental, physical, social and economic, independent of severity of disease and ability to recover. However, it depends very much on the patient’s own intention for control and personal circumstances whether shared decision making tends towards a result based on patient preference or the physician. The duty to reduce risks to a minimum remains independent of the fact of whether shared decision making and patient’s risk acceptance lay at the basis of the MIMVS intervention.

Even when the benefit–risk profile objectively favors MIMVS, the patient may prefer an incomplete transcutaneous solution by means of TMVR. Many circumstances including self-appreciation of their own life, social standing and insurance status all play a role. Shared decision-making outcomes prevail over scientific evidence and institutional data [69,70]. This could also be said to be a powerful form of patient preference bias.

Anyanwu [62] commented on differences in outcomes between institutions in response to the same treatment, and he proposes seven forms of possible selection bias in cardiovascular surgery, including Population Bias (level of economic development); Institutional Bias (type of funding, academic); Referral Bias (distance, insurance); Treatment Bias (high/low-volume surgeon—low-risks): Classification Bias (additional procedures); Survivor/Time Dependent Bias; Lead Time Bias (asymptomatic—very early disease stage); and Hidden Bias (Team. Logistic, subjective factors). It is paradoxical that effective risk control usually entails making choices to meet the appropriateness of the indication, and therefore this can be seen as the sensible application of selection bias. Patient-tailored risk profiling for mitral intervention is essential for minimizing risks. In addition, shared decision making may over-express patient preferences. Hence, applying these types of bias results in satisfied patients and good outcomes, but at the same time hampers objective comparisons of outcomes.

The NHR presents comparative results based on risk-adjusted mortality and a limited set of adverse events [8]. The short-and long-term results from the OLVG look good. However, selection bias may play a role as fewer patients with acute endocarditis and a EuroSCORE II > 9 were included. A confounding factor is that MIMVS was not a separately defined parameter in the NHR. Although it is known that in some hospitals MIMVS is the preferred approach, the data from the institutions allow only comparisons to be performed of clinical outcomes. The degree to which registry data are suitable to answer questions about surgical techniques, in particular the use of auxiliary technology, is limited. Despite many studies that included a large number of patients undergoing MIMVS, there is still debate regarding the superiority of a particular mini-access route and even conventional sternotomy [71]. A recent study which included the same mitral valve surgery data from the NHR concluded that based on an analysis of registry data there was no reason to assume superiority of MIMVS in general [72].

Registry data represent the results of heart team decision making and IRMP in a particular hospital, and therefore quantitative comparison reveals weak and/or best practices among participants [73]. To the individual patient, every high-impact event is the realization of a catastrophic risk. The reduction of such risks by means of a strong IRMP system is always meaningful and therefore contributes to best practice.

How surgeons shape and operate the best practice presented in the NHR remains concealed to outsiders. It is a part of the hospital’s internal quality control system. In our retrospective analysis of mortality, two of seven deaths were due to technical shortcomings, which we considered preventable (Table 2). Even though the absolute number for serious bleeding followed by re-exploration is low, in retrospect it appears that we could have performed better in five of the eight cases of excessive bleeding. Ko et al. described the MIMVS experience in 745 patients and reported a 30-day mortality of 1.2% and a stroke rate of 0.3% at 30 days [74]. Re-exploration for bleeding within seven days was the most common major complication. Ko’s paper is relevant as it adapts definitions of complications of the Mitral Valve Academic Consortium (MVARC) and reports the rate of freedom from any major complication < 30 days of 87.2%, and also the nature of these complications. Despite low and very acceptable adverse event rates for the high-impact complications, a root cause analysis and subsequent suggestions for learning and improvement are missing. However, clinical performance data are usually presented in a descriptive way only. Despite agreements on definitions, the interpretation of clinical outcome and risks in terms of adjusting MIMVS, the impact of IRMP remains underexplored. This was also the case in our team. We found that staff and residents only considered it valuable to discuss high-impact complications. The fact that serious bleeding usually turns out not to be lethal was considered a matter of fact, even though bleeding weighs heavily as a negative factor in the comparison with TMVR. Although POCMA was attempted, our dataset was small and the meaningful use of POCMA requires large datasets with a considerable number of events. PRISMA or FMEA/FTA work better in relatively small datasets which include fewer than 500 patients with a rather low number of a priori high-impact events and major complications. However, the investments for operating these risk management systems are high considering the scale of the hospital activities, which take place at the workshop level unlike in industry. Nevertheless, we can conclude that the rates of freedom from minor and major complications according to the MVARC definition are sound benchmarks for the effectiveness of IRMP.

This brings us to a practical question, why pay attention to the iceberg events as precursors of a catastrophe or high-impact event, when this analysis process is time-consuming, and at first sight cannot significantly decrease catastrophic complications? The answer is that IRMP systems and structural root cause analysis reduce avoidable pain, discomfort and suffering and further minimize risks of high-impact events. IRMP makes MIMVS competitive in terms of risks of pain and discomfort, and quick rehabilitation. This system can provide transparent input for shared decision making by means of concise, actual and precise risk information to patients. This type of risk information can shape the patient’s actual expectations of benefits and risks, create risk acceptance and controls fear when faced with the choice of full sternotomy, MIMVS or TMVR intervention. IRMP information should move from the back office to the front desk where the physician discusses the risks and benefits with the patient instead of sharing assumptions based on publications by other institutions.

Operating a quality control system is time consuming and demands input from other disciplines. It can be reasonably expected that the use of artificial intelligence to handle big data and assist learning will relieve this burden. By enabling selection and crimping the video footage and the stream of monitor data in OR and ICU, a more user-friendly application of PRISMA, POCMA and FMEA will become achievable. Machine learning is a promising methodology if large datasets are available for predicting outcome, and it may become a better tool to create risk-adjusted standards [63,64].

Is the use of new technology less time-consuming and does it contribute to better clinical outcomes in an MIMVS registry? Computational modeling of anatomy and function prior to the intervention reduces the likelihood of surprising findings and facilitates a planned and swift execution of the procedure. The availability of this information requires a preoperative process of investigation and questioning, which can be based on a 3-D print, image, or hologram. Intraoperative support is provided by means of augmented reality or a surgical robot. However, comparative studies on whether this technology significantly improves results and the MIMVS outcome are lacking, despite the occasional encouraging report. Therefore, the impact of surgical innovation must be primarily assessed at the institutional level by means of the critical appraisal of work processes and risk management. Safety for patients entails less risk and less pain and discomfort. Therefore, in order to assess the impact of innovations, it is essential to obtain information from an IRMP system, as innovations may introduce new risks and not meet expectations.

**Figure 2:**
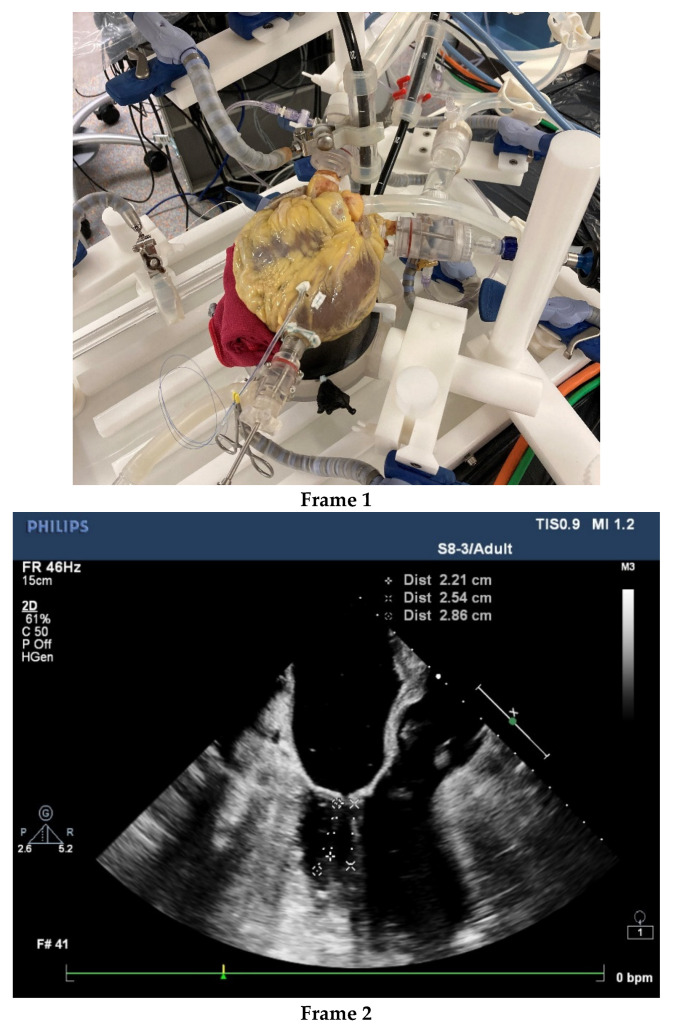

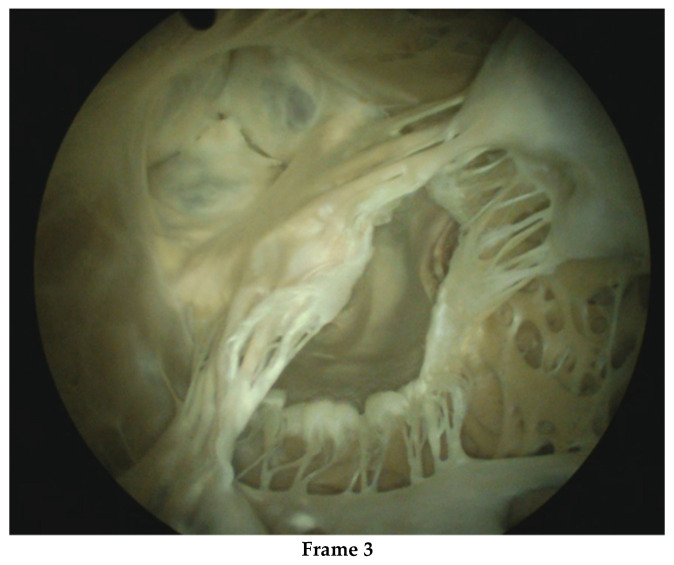
After thawing, the human cadaver heart is embalmed with a specialized solution of a very low toxicity. This embalmment ensures preservation, safe and user-friendly handling of the heart and tools and improves tissue quality, which allows for physiologic load of the heart, although protection measures always remain in place. **Frame 1**. provides an external view of the embalmed human cadaver heart. **Frame 2**. shows a TEE of the pulsating heart with an assessment of the diameter of the mitral valve diameter, the association with the aortic valve and length of chordae. **Frame 3**. presents a still of the videoscopic presentation of the left ventricle and partial left atrium.

The aforementioned imaging add-ons may simplify surgical duties and tasks despite their technical complexity, but these require specialized knowledge and computer competence. Computer/ robotic assistance also forces surgeons to reflect on the boundaries of their surgical proficiency and skill building. Control of the surgical surroundings has become far more complex than the operation itself. The proficiency of the surgical team and the institution should be critically examined. From admission to rehabilitation care, within a surgical group and the institution, minimal invasiveness must be the common interventional approach supported by adequate infrastructure, cost-effective logistics and educated and skilled workers. Surgeons are not infallible and, in our experience, some cases of bleeding and death were associated with suboptimal surgical performance. It remains a challenge to select surgeons for MIMVS and to train them effectively. Apart from learning-by-doing, simulators may be used to select candidates for MIMVS and accelerate proficiency, especially if human cadaver hearts are used [75,76,77]. See Figure 2.

Beating human cadaver hearts with intact arterial and venous connections provide an excellent real-life training platform. In prescreening, the human cadaver heart can be inspected and tested, to ensure that the heart is suitable for the test or teaching requirements. Such a platform can be placed in a cath-lab imaging environment including 3-D echocardiography. The setting is not only effective for testing devices, but also for assessing how much imaging information and device handling a trained operator can safely master. The platform may reveal complex usability issues relevant to organizing the work processes and safe learning of MIMVS by junior surgeons and to mastering new technology by advanced surgeons. Safe and fast MIMVS starts with excellent surgeons, and in our opinion technical innovation is no substitute for suboptimal surgical skills. In contrast, high-tech support for moderately competent surgeons may create serious additional risks, including the interruption of workflow [78].

From the point of view of ergonomics, we arrived at a crossroads where we must decide how much complexity and decision power a single MIMVS surgeon can handle by offering more technology, or how much decision power the surgeon should delegate to team members by adopting the computer-supported innovations, resulting in a split into operating and ‘commanding’ surgeons. Although less complex in terms of execution, the planning and predicting of the success of a TMVR procedure is more difficult, and it certainly benefits from advanced imaging and 3-D technology. In MIMVS, a competent surgeon can successfully deal with unexpected challenges due to their abilities learned through training and experience.

## 5. Conclusions

MIMVS can be conducted with low and very low surgical risk and can compete with TMVR based on interventional risk assessment and prognosis, even in functional MVR. In a shared decision-making process, the advice of the heart team must include knowledge of their own internal IRMP outcomes as well an explanation of how this information was generated. MVARC-based definitions of complications enable standardized rates for freedom from high-impact major and minor complications, which are parameters for the effectiveness of IRMP.

A national registry such as the NHR is a quantitative quality control tool for monitoring MIMVS outcomes, and one which also reflects best practices, including IRMP. A closer look at the results from the OLVG hospital showing low rates of catastrophic and high-impact events reveals that in retrospect some events could have been prevented. Despite knowing that catastrophic or high-impact events are preceded by many precursor or warning events, little attention was paid to the structural collection and analysis of these iceberg events. Root cause analysis methods such as PRISMA and FMEA/FTA are helpful, however, these methods require dedicated time and special expertise, while bureaucracy should be avoided. Nevertheless, machine learning and artificial intelligence can provide effective and objective event analysis platforms. From the point of view of the total number of risks to be managed in MIVMS, it is worthwhile to operate an IRMP system, not least to be able to reassure patients and reduce their fear of pain and discomfort. In MIMVS procedures, there seems more to be gained from attacking critical bottlenecks in patient management, varying from the administration of cardioplegia to the prevention of delirium. The ever-growing role of computer assistance may facilitate better surgery, but it will also create new risk-control challenges related to complexity, logistics and surgical competence. Based on analyses of the work processes, heart team decision making and the planning of TMVR may benefit from computerized imaging and modeling support.

However, a proper IRMP system is able to monitor, analyze and control new and known risks during the process of introducing innovations to MIMVS and TMVR. An IRMP system can also clarify expected and unexpected events. For these reasons, we recommend that on arrival at the MIMVS crossroads, priority should be given to investing in an IRMP system as this precedes the need for, and the evaluation of, surgical innovations included in a work process. A second question to be tackled at the crossroads relates to the application of new technology. At what level of complexity and dependency on non-surgical expertise of the conventional MIMVS should surgical tasks be divided between a commanding surgeon and an operating surgeon?

However, first, let us begin by finding, training and retaining excellent MIMVS surgeons.

## Figures and Tables

**Table 1:** Summary of perioperative variables in the MIMVS isolated mitral group.

2016–2020 *n* = 372	
120-day mortality	1.1%
1-year mortality	1.9%
Stroke	0.3%
Postoperative mitral insufficiency (mild or severe)	1.4%
EuroSCORE II (mean)	3.56%
Rethoracotomy	2.2%
Previous cardiac surgery	10.8%
Age > 75 years	20.4%

**Table 2:** Reoperation due to postoperative bleeding. ES II: EuroSCORE II *: adverse event (s), * d: postoperative day of reoperation after the initial surgery, LMWH: low molecular weight heparin.

Description	ES II	Preoperative	Perioperative	Postoperative	Discharge	Avoidable
Postoperative bleeding	1.84%			* d0		Y
Fragile patient with high-risk profile	6.9%	*		* d6		Y
Epicardial vein injury	0.89%		* d0			Y
Fragile 86 year-old patient under Warfarin	1.40%	*		* d2		N
Fragile patient with Lupus, previous right lobectomy and under Warfarin, LMWH and Aspirin	1.96%	*			* d22	Y
Spontaneous Pectoral muscular bleeding under Warfarin, LMWH and Aspirin	11.25%			* d5		?
Pectoral muscular bleeding	2.41%			* d2		?
Diagnostic error Minimally invasive mitral surgery undiagnosed ventricular rupture	42.68%	*	* d0			Y

**Table 3:** Postoperative mortality. ES II: EuroSCORE II, *: adverse event (s), †d: postoperative mortality in days after surgery, MOF: multiorgan failure, EF: ejection fraction, OHCA: out of hospital cardiac arrest, LV: left ventricle.

Description	Age	ES II	Preoperative	Perioperative	Postoperative	Discharge	Avoidable
Very high-risk case-bleeding and mitral annular rupture	66	12.47%	*	† d0			N
High-risk, delirium, MOF	77	5.08%			*	† d112	N
Heart failure, low preoperative EF	71	17.76%	*			† d21	N
OHCA 1 day after discharge	69	6.41%			*	† d9	Y/N
Surgery complicated by iatrogenic catheter perforation, MOF	79	5.89%		*	† d37		Y
Very high-risk re-redo	80	81.32%	*		† d20		N
MOF Papillary muscle rupture with preoperative undiagnosed LV wall perforation	78	42.68%	*	*	† d42		Y

**Table 4:** Summary of possible measures to prevent surgical complications/events.

Prevention of Adverse Surgical Events—Downsides
Preoperative adequate CT scan assessment—no evidence of cost-effectiveness Vascular closure device femoral—no evidence of cost-effectiveness Transapical Micro-invasiveness—limited indications Structural mini-invasiveness program—scale/specialists vs. generalists Specialized multidisciplinary heart teams—ineffective presence/time-consuming Atrial appendage closure—costs and selective indication Patient-tailored pre- and postop. anticoagulant therapy—uncertainty and difficult protocol Intercostal Block for pain relief—expensive/nerve injury Delirium detection—possible overtreatment and no evidence of cost-effectiveness Computer-assisted modeling and imaging—expensive/time-consuming Patient-tailored rehabilitation—time-consuming/no evidence

## Data Availability

Data are stored in the data repository and hospital information system of the OLVG Hospital. NHR registry data are available from the Annual Report, which can be downloaded as indicated in reference [8].

## Abbreviations

CVA: Cerebro—Vascular Accident, AI: Artificial Intelligence, FEA: Fault Tree Analysis, FMEA: Failure Mode and Effects Analysis, DOAC: Direct Oral Anti-Coagulants (DOACs), EEG: Electro Encephalo-Gram, ICU: Intensive Care Unit, IRMP: Institutional Risk Management Performance, MIMVS: Minimally Invasive Mitral Valve Surgery, MOF: Multi-Organ Failure, MVARC: Mitral Valve Academic Consortium, MVR: Mitral Valve Regurgitation, NHR: Netherlands Heart Registry, OHCA: Out of Hospital Cardiac Arrest, OLVG hospital: trade name of the previously named Onze Lieve Vrouwe Gasthuis, Amsterdam, The Netherlands, OR: Operating Room, POCMA: Phases of Care Mortality Analysis, PRISMA: Prevention and Recovery Information System for Monitoring and Analysis, RPN: Risk Priority Number, STS: Society of Thoracic Surgeons, TEE: Trans-Esophageal Echocardiography, TMVR: Transcatheter Mitral Valve Repair.
